# Organotypic heterogeneity in microvascular endothelial cell responses in sepsis—a molecular treasure trove and pharmacological Gordian knot

**DOI:** 10.3389/fmed.2023.1252021

**Published:** 2023-11-09

**Authors:** Audrey Cleuren, Grietje Molema

**Affiliations:** ^1^Cardiovascular Biology Research Program, Oklahoma Medical Research Foundation, Oklahoma City, OK, United States; ^2^Department Pathology and Medical Biology, University Medical Center Groningen, University of Groningen, Groningen, Netherlands

**Keywords:** sepsis, multiple organ dysfunction syndrome, endothelial cell heterogeneity, human studies, animal models, -omics, pharmacology, biomarkers

## Abstract

In the last decades, it has become evident that endothelial cells (ECs) in the microvasculature play an important role in the pathophysiology of sepsis-associated multiple organ dysfunction syndrome (MODS). Studies on how ECs orchestrate leukocyte recruitment, control microvascular integrity and permeability, and regulate the haemostatic balance have provided a wealth of knowledge and potential molecular targets that could be considered for pharmacological intervention in sepsis. Yet, this information has not been translated into effective treatments. As MODS affects specific vascular beds, (organotypic) endothelial heterogeneity may be an important contributing factor to this lack of success. On the other hand, given the involvement of ECs in sepsis, this heterogeneity could also be leveraged for therapeutic gain to target specific sites of the vasculature given its full accessibility to drugs. In this review, we describe current knowledge that defines heterogeneity of organ-specific microvascular ECs at the molecular level and elaborate on studies that have reported EC responses across organ systems in sepsis patients and animal models of sepsis. We discuss hypothesis-driven, single-molecule studies that have formed the basis of our understanding of endothelial cell engagement in sepsis pathophysiology, and include recent studies employing high-throughput technologies. The latter deliver comprehensive data sets to describe molecular signatures for organotypic ECs that could lead to new hypotheses and form the foundation for rational pharmacological intervention and biomarker panel development. Particularly results from single cell RNA sequencing and spatial transcriptomics studies are eagerly awaited as they are expected to unveil the full spatiotemporal signature of EC responses to sepsis. With increasing awareness of the existence of distinct sepsis subphenotypes, and the need to develop new drug regimen and companion diagnostics, a better understanding of the molecular pathways exploited by ECs in sepsis pathophysiology will be a cornerstone to halt the detrimental processes that lead to MODS.

## Introduction

Forming a barrier between the blood and underlying parenchyma, endothelial cells (ECs) cover the inner lining of all blood vessels. Together with circulating immune cells, they actively engage in the host response to bacterial and viral infections. While this is initially a localised process aimed at eliminating the pathogen and restoring tissue homeostasis, an excessive and dysregulated response can eventually result in sepsis (1). Despite improvements in patient care, there is currently still no effective treatment directly improving clinical outcome (2), and sepsis remains a global health problem with incidence estimates nearing 50 million cases per year and 11 million sepsis-related deaths reported in 2017 (3).

**Figure 1:**
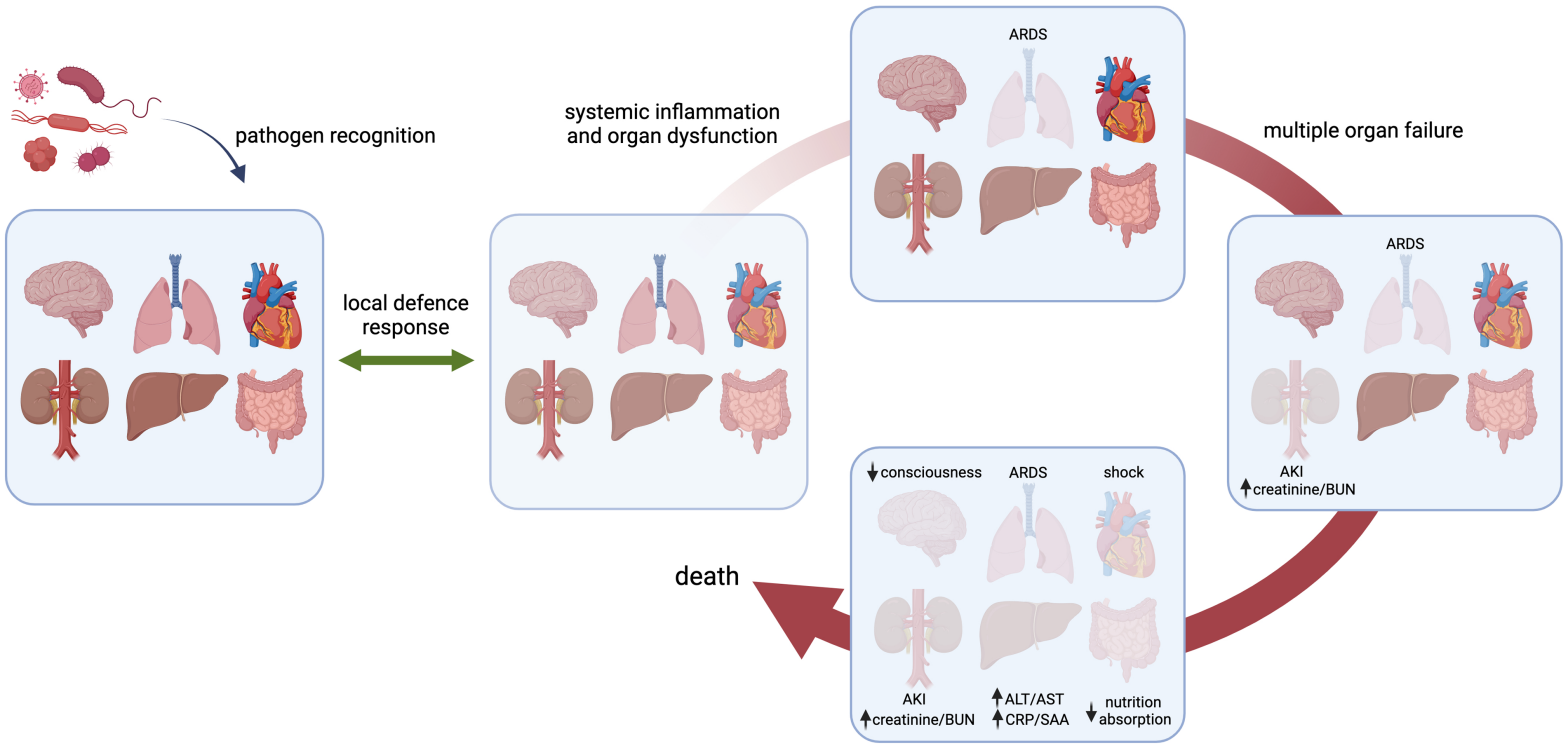
Schematic presentation of progression of organ loss in sepsis in response to an invading pathogen. Initially, the host senses the infectious microorganism that results in a local defence response to eliminate the pathogen and restore tissue homeostasis. (A dysregulated host response can lead to systemic inflammation and organ dysfunction, exemplified here for the lung, eventually leading to failure of multiple organs and death. Examples of specific organ failure as seen in the clinic are listed. The order and kinetics of organ systems affected are arbitrarily chosen and do not occur in a sequential fashion *per se*. ARDS, acute respiratory distress syndrome; AKI, acute kidney injury; BUN, blood urea nitrogen; ALT/AST, alanine transaminase/aspartate transaminase; CRP/SAA, C-reactive protein/serum amyloid A.

Progressive organ dysfunction is common in sepsis pathogenesis, leading to multiple organ dysfunction syndrome (MODS) when two or more organs are affected (Figure 1). In the clinic, this typically presents as diminished creatinine clearance, elevated blood urea nitrogen and oliguria/anuria; acute respiratory distress syndrome (ARDS); elevated bilirubin levels, dysregulated production of plasma coagulation factors and increased production of acute-phase proteins; malabsorption in the gut; lower consciousness; shock, cardiac dysfunction and/or thrombocytopenia and disseminated intravascular coagulation (4–7). Furthermore, patients with sepsis have systemically elevated levels of vascular cell adhesion molecule 1 (VCAM-1), vascular endothelial cadherin (VE-cadherin), EC-derived coagulation factors such as von Willebrand factor (VWF) and soluble thrombomodulin, as well as angiopoietin-1 and -2, thus strongly implying engagement of the endothelium in the body’s response in sepsis (8–12). Indeed, the microvasculature, which represents the largest surface of the vascular tree, is not only a main target but also a contributor to the sepsis-associated pathophysiological processes underlying MODS since it is the predominant site of leukocyte recruitment and blood vessel permeability, and is important for maintaining the haemostatic balance (5, 7, 13).

Given the strong dependence of ECs on their microenvironment, studying their role necessitates looking at their behaviour in an *in vivo* context, since enzymatic dissociation or culturing ECs leads to dramatic shifts in gene expression profiles (14–16). The organ- and microenvironment-specific behaviour also precludes the extrapolation of observations from one vascular bed to another without further validation (17, 18). This does not only hold true for changes observed under pathophysiological conditions, but also for studying pharmacological interventions aimed at the microvasculature (19). Although it is still technically challenging to study ECs *in vivo*, recent technological advances have created exciting new opportunities in this respect. Based on these developments we have chosen to focus this review on a combination of hypothesis-driven and unbiased -omics studies reporting *in vivo* endothelial responses in sepsis. While the former typically interrogate a single molecule or pathway, the latter provide a more comprehensive approach that has the potential to unveil previously understudied pathways important in sepsis pathology, and could thus lead to the identification of novel therapeutic targets.

We first provide a brief introduction on blood vessel and endothelial cell heterogeneity, as well as on relevant functions of the microvascular endothelium. We will then describe our current understanding of endothelial responses in sepsis and sepsis-related conditions based on studies performed in tissue samples from patients who died of sepsis, and in animals subjected to experimental sepsis, with a special focus on EC reactivity across organs. Both the differences and similarities in endothelial responses between tissues may provide opportunities for the design of biomarker panels that can be measured in blood or urine to determine the kinetics of microvascular engagement in response to infection. A better understanding of organ-specific molecular reactions of the microvascular endothelium in sepsis is also essential for a rational design of (combination) therapies that interfere with organotypic EC dysfunction. By providing a perspective that combines knowledge from the past with technological innovations of today, we hope the long-standing notion of sepsis being “the graveyard of pharmaceutical industry” (20) will become invalid.

## Endothelial cell heterogeneity

### Structural and functional heterogeneity

**Figure 2:**
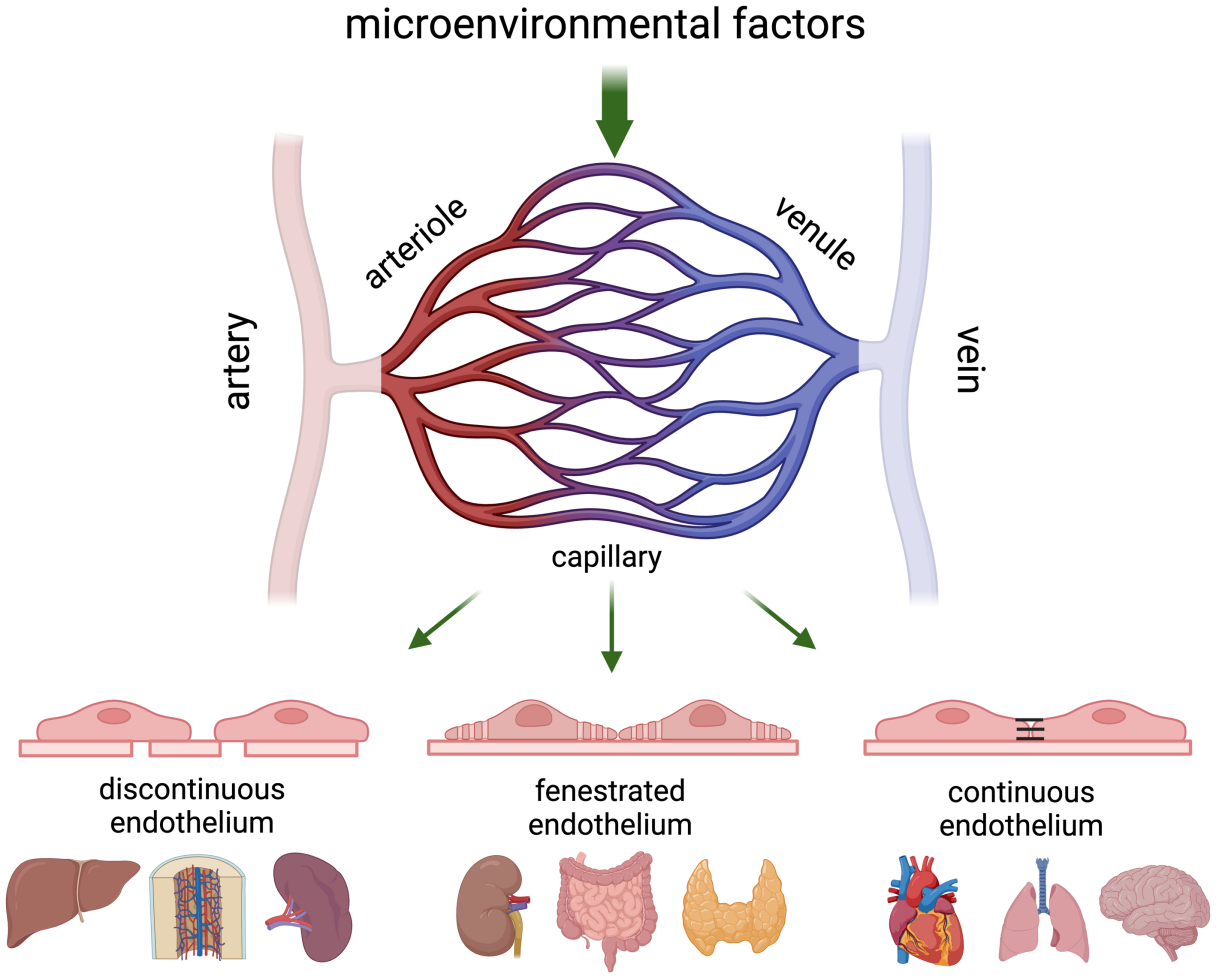
Schematic presentation of the gross architecture of blood vessels in an adult vertebrate. The cellular composition of blood vessels changes along the vascular tree where arterioles transport the blood from arteries into the tissue capillaries, and postcapillary venules transport it back to the bigger veins. Vascular permeability and leukocyte recruitment is predominantly regulated at the level of the capillaries and postcapillary venules, which show organ-specific structural differences based on inter-endothelial connections with continuous capillaries allowing the most controlled passage of blood and soluble components in the blood, and discontinuous capillaries allowing free passage. Although vessels are often categorised based on diameter, scRNA-seq studies have shown that there is a continuum of transcriptional states in ECs across the different branches of the vascular tree.

All blood vessels in the body consist of an endothelial cell monolayer that is in direct contact with the blood and supported by mural cells (smooth muscle cells in larger veins and arteries, pericytes in the microvasculature). The structural distinction along the different branches of the vascular tree is predominantly dependent on mechanical forces (shear stress), whereas structural differences within the microvasculature are mostly dictated by the needs of the underlying parenchyma (Figure 2). For example, the liver sinusoidal endothelium is highly permeable as it consists of discontinuous ECs that lack a basement membrane and have open fenestrae to allow free transfer of fluid, nutrients, and both small and large molecules (21, 22). Endo- and exocrine glands, intestinal mucosa, and dedicated microvascular segments in the kidney all contain fenestrated endothelium to accommodate efficient secretion into and filtration of the blood (23–26). Microvessels in the heart, skin and lung are less permeable and contain continuous, non-fenestrated endothelium hallmarked by the presence of caveoli and vesiculo-vacuolar organelles (VVOs). While caveoli assist in the passage of macromolecules from blood into tissue (27), VVOs are involved in macromolecular extravasation and typically located in the ECs of postcapillary venules from organs that are sensitive to permeability-increasing signals induced by for example, vascular endothelial growth factor (VEGF) and histamine (28).

The correlation between structure and function of the endothelium is also clearly illustrated in the blood-brain barrier, where adherens junctions and tight junctions form strong connections between ECs to avoid noxious stimuli present in the circulation from entering the central nervous system. Particularly the presence of tight junctions, which are composed of occludin and members of the claudin, zona occludens and junction-associated molecule families, limits transcytosis. Therefore, to enable selective transfer of solutes from the blood into the brain parenchyma, these ECs express many highly specialised transporters (29, 30).

### Molecular heterogeneity

As illustrated above, ECs need to tailor their form and function to the microenvironment they reside in. While this has long been recognized, understanding the observed heterogeneity at the molecular level was initially limited to studies evaluating specific genes and proteins in a low-throughput manner via histological approaches or the use of transgenic animals (31). Although crucial in laying the foundation for discerning the role of the endothelium in regulating immune cell recruitment and extravasation, barrier function and vascular permeability, and its contribution to coagulation, it was the introduction of unbiased -omics approaches that has greatly enhanced our knowledge on the molecular mechanisms underlying the structural and functional heterogeneity of ECs. Particularly high-throughput transcriptomics combined with improvements in methods to select ECs (32–34), EC-associated transcripts (16, 35–37) or luminal proteins (38, 39), has made it possible to evaluate the endothelium directly from its native *in vivo* environment in an unbiased manner (Table 1).

**Table 1:** Overview of studies evaluating organ-specific EC expression profiles.

Organ	Species	Method	Vascular/EC enrichment	References
Multiple	Mouse	Cell sort, bulk EC RNA-seq	Yes	(32, 33)
		TRAP, bulk EC RNA-seq	Yes	(16, 36, 37)
		TU-tagging, bulk EC RNA-seq	Yes	(35)
		scRNA-seq	No	(40)
		scRNA-seq	Yes	(41, 42)^a^
		scRNA-seq	Yes	(43, 44)
		scRNA-seq	No	(45)
		Proteomics luminal proteins	Yes	(38, 39)
	Human	scRNA-seq	No	(46)
Brain	Mouse	scRNA-seq	Yes	(47, 48)
	Human	snRNA-seq	Yes	(49, 50)
		scRNA-seq	Yes	(51)
Heart	Human	scRNA-seq, snRNA-seq	No	(52, 53)
		snRNA-seq	Yes	(54)
Kidney	Mouse	scRNA-seq	Yes	(55, 56)
		scRNA-seq	No	(57, 58)
		scRNA-seq, snRNA-seq	No	(59)
	Human	scRNA-seq, snRNA-seq	No	(60)
		scRNA-seq	No	(61)
Liver	Mouse	scRNA-seq	Yes	(62)
	Human	scRNA-seq	Yes	(63)
		scRNA-seq	No	(64)
Lung	Mouse	scRNA-seq	Yes	(65)
		Cell sort, bulk EC RNA-seq	Yes	(34)
	Human	scRNA-seq	No	(66)

a Re-analysis data Tabula Muris.

Despite being invaluable in establishing organotypic EC heterogeneity, it is important to note that these EC-enrichment methods do not allow the deconvolution of distinct endothelial subpopulations within a tissue. Instead, the data form an averaged endothelial expression profile per organ. This limitation has at least in part been overcome by the introduction of single cell and single nucleus RNA sequencing (RNA-seq), which has further improved our understanding of EC heterogeneity by providing increased cellular resolution. This has enabled identification of the EC compartment from organ-specific single cell RNA-seq (scRNA-seq) data based on the expression of known EC markers (41, 42, 45, 46, 52–54, 57–61, 64, 66). Furthermore, by performing scRNA-seq analysis on preselected ECs or vasculature-enriched samples (43, 44, 47–51, 55, 56, 62, 63, 65), transcriptional differences across EC subpopulations within organs and along the arterio-venous axis have been established as recently reviewed (24, 67–70), with available studies and datasets summarised in Table 1. The revealed molecular heterogeneity of the endothelium between, and even within organs, has further strengthened the conclusions that observations from one tissue or vascular segment cannot simply be extrapolated to the next.

## Endothelial contribution to (patho)physiology

Although our knowledge on EC heterogeneity has vastly improved in recent years, it is still not fully understood how heterogeneous responses in pathological conditions are controlled at the molecular level. Yet, their rapid responses to fluctuations in the local milieu and their unique anatomical position make ECs both an early target and contributor to many diseases, including sepsis (71). As such, the endothelium produces and secretes proteins into the circulation that could be used in the development of biomarkers, while on the other hand it makes them attractive targets for pharmacological interventions (72). In the following paragraphs, we will provide a brief overview of processes essential to the function of ECs in maintaining tissue homeostasis, discuss the impact of EC heterogeneity on these processes, and how they are perturbed under sepsis conditions.

### Leukocyte recruitment

Leukocyte recruitment from the vasculature into the parenchyma in response to inflammatory conditions as present in sepsis involves sequential leukocyte tethering, rolling, adhesion, and transmigration through the endothelial layer. Under normal conditions, ECs are covered by the glycocalyx, a layer consisting of proteoglycans, glycosaminoglycans, and incorporated plasma- and EC-derived proteins that protects the endothelium from directly interacting with blood cells (73, 74). In the presence of cytokines such as TNFα, interleukin (IL)-6 and IL-8 the glycocalyx can be degraded, thereby exposing (upregulated) adhesion molecules including P- and E-selectin as well as VCAM-1 and ICAM-1 (75–78). These adhesion molecules, in addition to inflammation-induced chemokines (e.g., MCP-1), recognize their cognate receptors on circulating immune cells, and guide leukocyte tethering and adhesion under pathological conditions (79, 80). Leukocyte transmigration on the other hand, is largely dependent on dynamics of EC adherens junctions and involve pan-EC molecules like PECAM-1 (also known as CD31) and junctional adhesion molecules, which also play an important role in regulating vascular permeability.

Nowadays it is widely accepted that the recruitment of (subsets of) immune cells into the parenchyma is a complex concerted action that takes place predominantly in the microvasculature, and that this process is dependent on the molecular make-up of the local tissue environment. For example, T lymphocyte recruitment into the brain in response to experimental encephalomyelitis has been shown to be dependent on the local presence of laminin α4 (81), while leukocytes expressing the chemokine receptor CCR10 preferentially home to skin endothelium, and CCR7-positive leukocytes migrate into secondary lymphoid organs (82). Furthermore, early studies using radiolabelled antibodies specific for VCAM-1 and ICAM-1 have shown that constitutive expression of these 2 adhesion molecules is different across organs, with ICAM-1 levels being higher than VCAM-1 in brain and heart (83). These observations have been recently confirmed in bulk RNA-seq data of EC transcripts after translating ribosome affinity purification, which in addition showed the opposite result for kidney and to a lesser extent lung endothelium (16). Subsequent single cell RNA-seq data identified aerocytes (a specific EC subset in the lung) as the main ICAM-1-positive capillary cell (65), while the cerebral cortex contains a specialized postcapillary EC population characterized by constitutive expression of adhesion molecules, including ICAM-1 (47). Not surprisingly, for both organs these EC subsets have been shown to be the preferred site of immune interactions and leukocyte transmigration (47, 65).

### Vascular integrity and permeability control

As mentioned, microvascular ECs have distinct morphological characteristics reflecting the permeability control required to exert their physiological function (27, 30). In addition to claudins and occludin that encompass tight junctions, VE-cadherin (encoded by *CDH5*) has been well-established as a gate-keeper of endothelial integrity in which EC–EC interactions are being formed by adherens junctions (84, 85). The dynamic expression of VE-cadherin is dependent on its phosphorylation status, with phosphorylation via VEGFR2 (*KDR*) or PECAM-1 leading to its endocytosis and degradation, resulting in increased vascular permeability (86).

**Figure 3:**
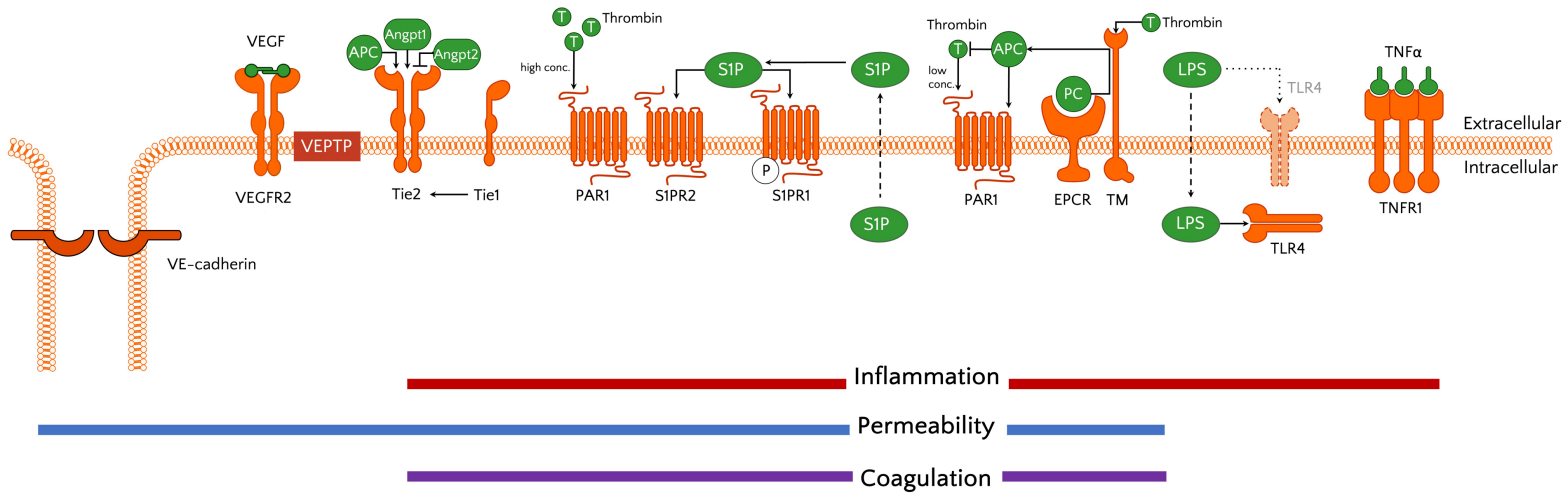
Schematic showing molecular systems important in endothelial (patho)physiology. Activation of receptors provides the microvascular ECs a pro-inflammatory, permeable, and coagulant status. The receptors and their ligands include examples discussed in the text, yet are not all encompassing. Of note, although TLR4 is traditionally known as a transmembrane receptor in myeloid cells, previous studies have demonstrated its intracellular location and function in ECs (87, 88). The reader is referred to Luxen et al. (90) for more detailed information on the signal transduction cascades that are activated by the receptors shown. APC, activated protein C; angpt, angiopoietin; EPCR, endothelial protein C receptor; LPS, lipopolysaccharide; PAR1, protease-activated receptor 1; PC, protein C; S1P(R), spingosine-1-phosphate (receptor); T, thrombin; Tie, tunica intima endothelial kinase; TLR, Toll-like receptor; TM, thrombomodulin; TNF(R), tumour necrosis factor (receptor); VEGF(R2), vascular endothelial growth factor (receptor 2).

Stabilization of the microvasculature is further influenced by the receptor tyrosine kinase TIE2 (*TEK*) and its ligands angiopoietin (ANGPT)-1 and ANGPT-2 (Figure 3). In quiescent endothelium, locally produced ANGPT-1 binds TIE2 and can subsequently activate 2 distinct intracellular signalling cascades that lead to stabilization of EC-EC junctions via VE-cadherin and suppression of inflammation-induced EC activation (91, 92). When ECs become activated they release Weibel–Palade bodies that contain high concentrations of ANGPT-2 (93). This rapidly tips the ANGPT balance in favour of ANGPT2, which via competitive binding to TIE2 leads to destabilization of the EC layer, a process that also involves the orphan receptor TIE1 (94). The ANGPT-induced destabilization furthermore enables a third well-studied molecular system important for vascular permeability: the VEGF pathway, in which VEGF interacts with VEGFR2, thereby leading to increased vascular leakage (95).

In addition to these 3 main pathways regulating vascular permeability, other proteins have been identified to contribute to this process. Most notably is vascular endothelial phosphatase VE-PTP (*PTPRB*), which controls barrier function through its substrates that include VE-cadherin, TIE2 and VEGFR2 (96–98). Furthermore, sphingosine 1-phosphate (S1P) regulates endothelial integrity via binding to its G-protein coupled receptor S1PR1 that is expressed on ECs, thereby stabilizing adherens junctions (99, 100) (Figure 3). Interestingly, S1P binding to S1P receptor type 2 (S1PR2) was shown to be associated with increased permeability in conditions of acute inflammation (101).

Studies using conditional knock-out mice lacking endothelial VE-cadherin showed distinct patterns of organotypic microvascular leakage, with increased permeability in the heart and lung, while skin and brain vessels were not affected by loss of VE-cadherin (96). This could possibly be explained by differences in basal expression levels, as RT-qPCR and EC-enriched RNA-seq data showed that *CDH5* is more abundant in the lung and heart as compared to kidney, brain and liver (16, 102). Besides organotypic differences in expression of genes important for maintaining vascular integrity, heterogeneous phenotypes can also exist within tissues. For example, it has been shown that in quiescent conditions, VE-cadherin contains phosphorylated residues in veins but not arteries, which might prime the protein for rapid internalization under inflammatory conditions to allow the required increase in vascular permeability (86). In addition, the tight junction protein claudin 5 (*CLDN5*) is highly expressed in the brain as compared to the other organs, while a recent scRNA-seq study showed that the decrease in CLDN5 levels along the arteriovenous axis in the brain inversely correlated with histamine-induced vascular leakage (103). Similarly, ANGPT-2 is barely detectable in murine renal arterioles and postcapillary venules, while it is abundantly expressed in the glomerular microvasculature (19). Interestingly, although this ANGPT-2 expression in glomeruli coincides with high levels of TIE2, relatively low levels of ANGPT-1, and robust expression of VEGF and VEGFR2—a combination that is normally associated with promotion of barrier degradation and neovascularization—this vascular segment is not particularly leaky nor proliferatively active (19).

### Endothelial regulation of haemostasis

Under physiological conditions, ECs have an anticoagulant phenotype as they express tissue factor pathway inhibitor (TFPI), thrombomodulin (TM) and the endothelial protein C receptor (EPCR; *PROCR*) (104, 105). Their anticoagulant property is further supported by the presence of fibrinolytic proteins including tissue-type plasminogen activator (tPA) and the plasminogen activator inhibitor PAI-1 (*SERPINE1*). Endothelial activation causes a procoagulant shift and leads to the release of VWF, which is required for platelet binding and activation that enhances thrombin formation. Although thrombin is mostly known as a procoagulant factor, it can also serve as an anticoagulant via binding to TM to activate protein C (leading to the formation of activated protein C, or APC), a process that is further facilitated by EPCR (106) (Figure 3). In addition to its anticoagulant function, APC also exerts anti-inflammatory and barrier protection properties (107–109), and has therefore gained a lot of interest in the sepsis field as a target for pharmacological interventions in the past (see below). Besides activating protein C, thrombin can also bind and activate protease-activated receptors (PARs), with binding to PAR1 playing a role in regulating microvascular permeability (110). Additional crosstalk between regulation of haemostasis and vascular integrity also takes place via ANGPT-2, which was recently shown to inhibit TM-mediated formation of APC (111).

Endothelial heterogeneity with respect to coagulation factors has received substantial attention, with studies in the 1990s already showing distinct expression patterns for EPCR, TFPI and tPA across organs as well as along the vascular tree [recently reviewed in (31)]. Similarly, VWF expression has been shown to be particularly high in large blood vessels, but low in capillaries (31, 112, 113). VWF serves as a carrier protein for coagulation factor VIII (*F8*), and in the past decade it has become evident that FVIII is also synthesized in the endothelium, particularly in the liver and kidney (114, 115). These initial organotypic observations have since been validated in numerous studies, including those using scRNA-seq analyses such as the Tabula Muris Consortium (40). Evaluation of *VWF*/*F8* expression levels across different organs in this dataset showed that lung also contains a specific EC subpopulation that expresses *F8*, but interestingly these ECs do not express *VWF*. In line with this, a recent scRNA-seq analysis of alveolar capillary endothelium showed that 2 different EC types (aerocyte vs. “general” capillary ECs) each produce their own unique set of pro- and anticoagulant factors. Whereas expression of TFPI and PAI-1 was restricted to aerocytes, general capillary ECs expressed VWF and tPA (65). These data suggest a division of labour, where different ECs serve different functions in regulating haemostasis, which could potentially also explain the observations regarding *F8*/*VWF* expression in the lung (40).

## Microvascular engagement in multiple organ dysfunction syndrome in sepsis patients

Multiple organ dysfunction syndrome in sepsis patients is a complex disorder resulting from an aberrant host response to pathogen invasion, characterized by a derailed immune response leading to organ injury (116) (Figure 1). The endothelium actively engages in the establishment of MODS, as evidenced by clinical studies reporting increased circulating levels of EC-derived proteins including soluble E-selectin, VCAM-1, VE-cadherin, PECAM-1, VEGFR1, ANGPT-2, IL-8, VWF, TM, tPA, PAI-1, and glycocalyx constituents in sepsis patients (8–11, 117–123). Also in the human endotoxaemia model (124), a consistent rapid increase in many of these plasma markers were established after LPS administration, strongly suggesting a direct link between pathogen exposure and EC response (119, 125, 126).

Although important for diagnostic purposes, changes in EC-derived proteins present in the circulation do not provide direct information on the extent of organ-specific microvascular contributions. Being able to address the latter depends directly on tissue samples, yet studies reporting molecular and functional behaviour throughout different vascular beds have been scarce and are typically limited to those evaluating samples of patients who succumbed to sepsis. Nevertheless, histological analyses have shown the presence of immune cells in the periportal regions of the liver and kidney glomeruli (127, 128), and neutrophil infiltration in the lung has long been considered an important contributor to sepsis-related ARDS (129, 130). In addition to neutrophils, the number of macrophages and B- and T-lymphocyte are also increased in lungs of patients who died of *Neisseria meningitidis* septic shock (131). A direct comparison between lung and heart samples of these patients revealed that the influx of several leukocyte populations was 30–80% higher in the lung as compared to heart, whereas the brain was devoid of enhanced leukocyte recruitment (132). Despite this latter, haemostatic abnormalities have been observed in brain samples of sepsis patients, with areas displaying hypercoagulability as well as haemorrhaging, indicative of consumptive coagulopathy (128). In addition, enhanced coagulation has also been noted in the kidney, as illustrated by fibrin depositions in glomeruli and peritubular capillaries in a subset of sepsis patients (127, 128).

Besides evaluating cellular contributions, immunohistochemical analyses indicated increased levels of MCP-1 and PAI-1 in the alveolar space in the lung, likely expressed by alveolar capillary ECs, whereas in heart these proteins were upregulated and co-localized in the arteriolar microvascular segment (132). Furthermore, evaluation of kidney biopsies of septic patients for markers known for their role in vascular permeability regulation showed a decrease in ANGPT-1 levels while ANGPT-2 was increased and TIE2 remained unchanged. At the same time, expression of VEGF and VEGFR2 was also reduced (102). These changes are all expected to contribute to kidney failure associated with sepsis.

In a recent study, microarray analysis was performed in tissue samples of meningococcal septic shock patients, which provides an opportunity to evaluate transcriptional changes in an unbiased manner and determine differences and similarities in organotypic responses (132). Although all organs included in the study showed changes under septic conditions as compared to samples from non-inflammatory origin, the number of differentially expressed genes was highest in kidney, followed by lung, heart, liver, and was lowest in the spleen. Not surprisingly, many of these genes were related to the host’s inflammatory response. In addition, a decrease in transcripts associated with metabolism and energy production was also observed across multiple organs (132). Interestingly, short-term changes in metabolism can be beneficial to the host as a temporal increase in energy is required to eliminate the pathogen. However, uncontrolled and prolonged disruption of the metabolic balance is detrimental since at the same time energy should be preserved because of poor nutritional input (133).

While studies based on human tissue biopsies have been informative, interpretation of results is difficult as they are often performed on bulk tissue, which precludes identification of the cell-specific contribution to the observed molecular changes. Even for histopathological and immunohistochemical analysis, correlating spatiotemporal cellular and molecular changes in the microvasculature to clinical symptoms is challenging. This is partly due to the intrinsic heterogeneity in the patient population itself (age, underlying co-morbidities, *etcetera*), and also because it is often unknown when the patient got infected prior to presentation in the hospital (see Challenges below). It thus remains elusive whether EC responses occur prior to the establishment of MODS, or whether EC activation forms an integral part of its pathogenesis. Therefore, animal models are invaluable to provide a rationale for validation studies in the sparse clinical material available to date as they recapitulate processes underlying sepsis and allow for detailed cellular and molecular analyses in an organ- and cell type-specific manner over time.

## Microvascular engagement in animal models of sepsis

### Animal models of sepsis

Non-human primates are highly similar to humans in their anatomy and physiology, as well as their hemodynamic and cytokine responses to infection. Also the ability to provide supportive care similar to that in septic patients makes them an important model to study sepsis, and as such they have provided crucial insights in disease aetiology (134–136). Other larger animals including pigs and sheep have also been used as they too share many aspects of sepsis that are similar to humans (134). However, from an economical and feasibility perspective, mice are much more attractive due to the availability of genetically engineered animals and low costs. Most importantly, the relative ease of experimental models allows a more reproducible evaluation of sepsis pathology. Although the use of mouse models in sepsis research has been a topic of debate (137, 138) as they do not fully recapitulate the clinical complexity, they have significantly contributed to our molecular understanding of the host response to infection and form the cornerstone of translational research.

The most frequently used models to induce a sepsis-like phenotype are based on the administration of either a toxin (e.g., lipopolysaccharide; LPS) or viable pathogen, or by breaching an endogenous protective barrier such as done in the caecal ligation and puncture (CLP) model (139–141). Whereas LPS is often used because of its technical ease and reproducibility, it has also been criticised for not capturing the intricacies of sepsis. On the other hand, CLP results in a polymicrobial infection, leading to a response that is more representative for sepsis (142). Although considered the “golden standard,” it is more variable and the severity of disease is highly dependent on the experimental parameters. Despite these differences, both models display signs of MODS as indicated by an increase in serum creatinine and blood urea levels reflecting renal dysfunction, AST and ALT levels indicating hepatocellular injury, and lung myeloperoxidase representing increased neutrophil infiltration. In addition, these models lead to a decrease in body temperature and heart rate, and metabolic derailment of organs has also been reported in both the CLP model and LPS-induced endotoxaemia (143–146).

For the remainder of this review, we focus where possible on studies that reported sepsis-induced changes in more than one organ to best reflect human MODS. Moreover, this circumvents limitations in interpretation of combined results from different studies which would be complicated due to methodological variations in execution of experiments, such as the use of different dosages and administration routes of LPS, differences in experimental execution of the CLP model, and/or differences in the time frame in which molecular and functional changes were assessed. We will first discuss results from hypothesis-driven studies that directly evaluated molecules involved in leukocyte recruitment, regulation of vascular permeability, and maintenance of the haemostatic balance. This will be followed by an overview of animal studies that used unbiased transcriptomics approaches in order to get a more comprehensive overview of the vascular bed-specific changes that occur during, and contribute to, sepsis pathology.

### Microvascular engagement in animal models of sepsis—leukocyte recruitment

EC activation in sepsis can occur via the LPS-induced Toll-like receptor (TLR)-4 signalling cascade, interaction with circulating cytokines, and via changes in blood flow leading to differences in shear stress (147, 148). Histological analyses showed that leukocytes, predominantly neutrophils, infiltrate lung, and to a lesser extent liver and intestine, with the lowest neutrophil content observed in kidney and heart 4 h after LPS exposure (149). In the polymicrobial CLP model of sepsis a similar increase in neutrophil accumulation occurred in major organs including lung, liver and kidney, which lasted until at least 24 h after surgery (150–152). Despite the comparable neutrophil influx across these organs, formation of neutrophil extracellular traps (NETs), which has been associated with loss of organ function (153), was most prominent in lung and least in kidney (154).

In general, endotoxaemia models all result in an upregulation of E-selectin, P-selectin, ICAM-1 and VCAM-1 (18, 155–158). As expected, induction of gene expression occurs early, within 4 h after LPS exposure, and with the exception of E-selectin in the liver, all these changes were still present after 24 h (18, 157, 158). It is important to note that lung showed the least increase in expression as compared to other organs tested, which might relate to the relatively higher abundance of these proteins under basal conditions (157, 158).

In line with these observations, a previous study of *N. meningitidis* bacteraemia in pigs also showed increased levels of E-selectin, ICAM-1 and VCAM-1 in kidney, liver and lung with results in the latter being the most modest (159). *Escherichia coli* infection in mice identified the heart as the least responsive organ regarding the up-regulation of VCAM-1 as compared to changes in the lung, kidney and liver (160). Also in the CLP model, increases in E- and P-selectin occurred early, after 6 h (161), and their expression remained high in brain, heart and lung up to at least 24 h after sepsis induction (18, 162). Effects of CLP-induced sepsis on ICAM-1 and VCAM-1 expression were less prominent at these early time points in these organs. Wen et al. (163) showed that 14 days after CLP surgery ICAM-1 levels in kidney were elevated. However, it was not investigated whether this late stage increase in expression occurred in other organs as well.

Besides differences in expression levels across organs, immunohistochemical analysis of the lung and kidney indicated that 16 h post-CLP surgery E-selectin was primarily present in the bigger vessels in the lung (164). In the kidney, VCAM-1 was predominantly induced in arterioles whereas ICAM-1 was most abundant in glomeruli and peritubular capillaries (164).

Together these data indicate that spatiotemporal changes are unique to each organ and disease model, suggesting specialized functions and recruitment of potentially different (subsets of) immune cells across organs as well as different branches of the vascular tree within organs (158, 165, 166). Despite some differences in results between studies that were likely due to differences in pathogenic stimulus, in general selectins appear to respond more strongly than VCAM-1 and ICAM-1.

### Microvascular engagement in animal models of sepsis—increase in vascular permeability/leakage

Endotoxaemic mice display increased permeability in the kidney, lung, heart and spleen at 6 h after LPS administration (101, 149). For kidney and lung, this increase sustained at least until 24 h post-LPS, whereas this was not the case for heart and spleen (158, 167). Collectively, these studies indicated that there was significant leakage in main organs in the first 24 h after LPS exposure, although data on vascular leakage in the liver was inconclusive at the 6 h timepoint. Interestingly, LPS did not affect cerebrovascular permeability. On the other hand, CLP surgery increased vascular permeability in all major organs, including the brain (168, 169). Mice infected with *E.coli* or *Staphylococcus aureus* to simulate peritonitis or pneumonia, respectively, also displayed microvascular leakage in the kidney, lung, liver and heart at 6–7 h after infection (160, 170). The brain was not assessed in these latter studies.

The molecular changes underlying the observed increase in sepsis-induced vascular permeability are predominantly regulated via VEGF/VEGFR and angiopoietin/TIE2 receptor interactions (30). Although VEGF is not produced by ECs, it has a major impact on their function via binding to the EC-expressed VEGFR1 (*FLT1*) and VEGFR2 (*KDR*). Changes in VEGF expression under septic conditions showed a spatiotemporal expression signature. For example in the kidney, LPS caused an increase in VEGF mRNA levels 4 h post-LPS, while after 8 h a significant reduction was observed and levels were normalized 24 h after LPS administration (102). Protein levels followed this kinetics, though at 24 h after LPS challenge VEGF protein in kidney remained reduced compared to control conditions (158). In lung, mRNA decrease only occurred later in time, at 8 h after LPS exposure, and remained low until at least 24 h post-LPS, coinciding with reduced protein levels at 24 h (102, 158). In heart on the other hand, VEGF protein levels were significantly increased in the early hours of endotoxaemia, and returned to basal levels at 24 h (158). In contrast to VEGF, VEGF receptors are expressed by the endothelium. In endotoxaemia, only in lung the expression of VEGFR1 and VEGFR2 decreased and remained low until 24 h post-LPS. In kidney, expression of neither receptor was significantly affected in time. Also in mouse CLP-sepsis, VEGFR2 expression remained constant in the kidney, while it decreased in a time-dependent manner in heart, liver and lung (171).

Regarding the angiopoietin/TIE2 axis, loss of TIE2 expression occurred in all major organs at 4–8 h after LPS exposure, with the kidney and lung being most affected (157, 172). These changes coincided with a transient decrease in ANGPT-1 levels in kidney at 8 h, while in lung ANGPT-1 was already reduced after 4 h, and remained low until at least 24 h after LPS administration (102). At the same time, ANGPT-2 levels were increased at the 4 h and 8 h timepoint in both organs, but normalized after 24 h. In line with these data from LPS studies, ANGPT-1 levels were also downregulated in CLP-induced sepsis (171). However, in contrast to LPS, CLP resulted in a decrease in ANGPT-2 in heart, kidney, liver, and lung between 12 and 24 h after surgery.

Given its importance in adhesion junctions, VE-cadherin is also often evaluated with regard to changes in vascular permeability, and endotoxaemia has been shown to result in significantly decreased levels in the lung (164). Also in the CLP model, a reduction in VE-cadherin was present in the heart, lung, kidney and liver, which was associated with increased vascular leakage in the latter two (173).

Overall, it can be concluded that there is a general increase in microvascular permeability across different experimental sepsis models, though there is variation in the kinetics and extent of leakage between organs, as well as variation in the responses observed in specific pathways associated with vascular barrier function.

### Microvascular engagement in animal models of sepsis—pro-coagulant status

Dysregulation of the haemostatic balance is a common feature of sepsis, which can lead to disseminated intravascular coagulation contributing to the development of organ dysfunction (174). This procoagulant shift is reflected by increased levels of circulating thrombin-antithrombin complexes, tissue factor, PAI-1 and D-dimer levels (175), with the latter indicating fibrin degradation. LPS-induced endotoxaemia results in fibrin depositions in the liver, lung and kidney, where particularly bigger vessels and a subset of capillaries were affected in liver and lung 8 h post-LPS (175, 176). In kidney, fibrin was present in all microvascular segments at 24 h after LPS exposure (177). In a rat LPS model, fibrin deposition was reported to occur in liver capillaries, glomeruli and peritubular capillaries but not in the lung (178). However, they noted that LPS-induced endothelial injury was dependent on the dose and duration of LPS treatment. Finally, fibrin deposition was also observed in the liver after CLP-induced sepsis (176).

The procoagulant shift of the endothelium is greatly influenced by the downregulation of anticoagulant proteins such as TFPI and thrombomodulin (TM). For example, in response to LPS TFPI mRNA levels decreased in heart, kidney, and lung at the 8 h timepoint, with levels returning back to normal within 24 h (179). TM in rat liver and lung capillaries was strongly reduced within 2–4 h after LPS exposure, and a similar trend was observed in kidney peritubular capillaries, although TM expression in glomeruli remained high (178).

PAI-1 inhibits fibrinolysis, and in line with the previously mentioned increase in plasma PAI-1 levels, LPS also upregulated its local expression in heart (mostly in capillaries), kidney, lung and brain (predominantly in venules). Increase in PAI-1 levels was highest in heart, whereas brain and lung showed only a modest increase (158, 179). Although the origin of procoagulant tissue factor has been controversial (175, 180), a (transient) increase in expression was observed across multiple organs in a rabbit LPS model with the highest induction in brain, kidney, and lung at 2–3 h after LPS exposure (180). In mice, on the other hand, LPS caused increased TF expression in kidney and lung, but not in liver, heart or brain (101, 181). Interestingly, the observed increase in kidney appeared to be almost completely regulated via S1PR2 signaling, whereas changes in the lung were only partly regulated by S1PR2 (101), indicating an additional connection between coagulation and vascular barrier protection, besides the protein C pathway (107–109).

In summary, these results indicate that rapid increases in tissue factor and PAI-1, together with decreased levels of anticoagulant proteins such as TFPI and TM, contribute to a procoagulant shift that can lead to fibrin deposition. Changes occur primarily at the level of the microvasculature, although distinct organotypic differences exist.

## Use of -omics approaches in sepsis research

Based on the previously discussed hypothesis-driven studies, direct comparison of EC behaviour across organs revealed organ-specific differences in kinetics of molecular changes, yet literature on spatiotemporal responses of ECs is scarce. In addition, a big gap in knowledge exists to date regarding the location of molecular changes in microvascular branches in organs in response to sepsis conditions.

Unbiased approaches have become one of the most powerful tools to evaluate molecular characteristics of cells and organs in a systematic manner, and have provided valuable information on changes occurring in biological processes under pathophysiological conditions. Especially the application of single cell RNA-seq and spatial transcriptomics to help unravel protein–protein interactions important for identifying novel pathways contributing to disease pathogenesis, is eagerly awaited as this could enable the identification of potential diagnostic and prognostic biomarkers, as well as targets that would facilitate the development of therapeutics (182). The following section will discuss studies that used -omics analyses in order to get a better understanding of (organotypic) sepsis pathophysiology, and the contribution of the endothelium in this process. We will particularly focus on studies performing unbiased RNA-seq analysis of mRNA species in intact tissue (bulk RNA-seq), mRNAs bound to actively translating ribosomes isolated from endothelial cells specifically using a translating ribosome affinity purification (TRAP) approach followed by RNA-seq, and studies utilizing single cell RNA-seq that allows the evaluation of gene expression profiles at the individual cell level. We refer the reader to (31, 183, 184) for more information on these different techniques and their application in studying the vasculature.

### Organotypic changes in sepsis: bulk RNA-seq

One of the first studies using an unbiased approach to evaluate sepsis in an organ-specific way employed a rat CLP model, and identified subsets of genes that had both shared and unique expression patterns across different tissues that were temporally regulated (185). As expected, these included genes involved in inflammation and coagulation, but in addition they identified several transcripts that were not previously linked to sepsis-induced responses. For example, downregulation of genes known to be related to maintaining extracellular matrix (ECM) was observed in the lung and spleen, which suggests inadequate tissue repair. Furthermore, changes in transcripts important for antioxidant defence mechanisms were present in multiple tissues, including decreased levels of several members of the glutathione S-transferase gene family, of which glutathione S-transferase pi has been shown to play a role in regulating vascular permeability (186). Finally, they identified prominent alterations in genes associated with lipid metabolism across organs, and it is now well-established that lipid dysregulation in sepsis can affect the immune system and regulate the immune response by clearing bacterial toxins, reduce inflammation and inhibit the expression of adhesion molecules (187).

Many of these initial observations based on microarray-based data have since been confirmed using RNA-seq analysis. A recent study using a murine CLP model also identified significant changes in lipid metabolism and ECM remodelling in at least 2 organs, in addition to the expected (early) effects on the immune system and haemostasis (171). Metabolic dysregulation in the liver was already affected 6 h post-CLP, and changes sustained for at least 24 h. The kidney and lung also showed significant metabolic alterations at 12, respectively, 24 h after surgery, whereas the metabolic processes in the heart did not appear to be affected (171). This study furthermore validated the decrease in ECM genes in the lung, but interestingly found a CLP-induced overrepresentation of these transcripts in the liver. Overall, the number of significant differentially expressed genes for each organ was highest 12 h after CLP, with only the heart being almost completely normalized 24 h post-surgery.

### Organotypic changes in sepsis: EC-specific RNA-seq

Together, these studies illustrate spatiotemporal changes in gene expression patterns in sepsis in an unbiased way (171, 185). However, although they indicated a potential role for the endothelium in sepsis-related pathology, it must be noted that these studies used bulk tissue RNA for their analyses. The importance of studying transcriptional changes in a specific cell type, particularly those that, like ECs, are present in organs in low numbers, was initially shown using a translating ribosome affinity purification (TRAP) approach to determine EC-specific changes in an endotoxaemia model (16, 37). Similar to the apparent lack of CLP-induced changes in the brain in the rat CLP study (185), short-term LPS exposure in the mouse also resulted in minimal effects in the brain based on bulk tissue RNA-seq (16). However, when specifically evaluating expression profiles of the endothelial cells in the brain, the number of genes significantly affected was much higher and remarkably similar to those observed in other organs. Furthermore, with the exception of the brain, other target organs including the kidney, liver, lung and heart showed fewer EC-enriched genes to be upregulated than downregulated after LPS treatment, with the upregulated genes displaying a high degree of tissue-specificity. On the other hand, EC-enriched genes with reduced expression in LPS-treated mice were more commonly shared between organs (16).

EC-TRAP studies have confirmed many of the previously discussed organotypic alterations in expression levels of endothelial genes involved in leukocyte recruitment, cell junctions, and haemostasis (16, 37). In addition, gene ontology analyses based on transcripts significantly affected by LPS have indicated a consistent overrepresentation of genes involved in the regulation of metabolism across tissues. This has not only been observed after short-term LPS exposure, but a timecourse evaluating early and late inflammatory responses as well as changes occurring during the resolution phase also showed unique spatiotemporal expression patterns of metabolic genes (37). For example, brain EC-specific glycolysis genes were upregulated by LPS with expression levels remaining high throughout the course of inflammation progression and resolution, while glycolytic genes in ECs of the heart were not affected. In lung endothelium, the key glycolytic enzyme *Pfkfb3* showed a more dynamic pattern over time, with only a temporary rise in transcript levels. Targeting dysregulated EC metabolism has recently (re)gained increased interest as a therapeutic strategy for sepsis (188, 189), and it is worth noting that several studies have shown beneficial effects of PFKFB3 inhibition in acute sepsis-induced lung injury models (190, 191).

Besides evaluating transcript levels, recent studies have used *in vivo* biotinylation combined with proteomics to characterize the luminal surface of vascular cells (38, 39). These studies have confirmed organotypic differences in surface proteins under basal conditions as well as those induced by *S. aureus* infection. Not surprisingly, proteins that changed across multiple organs due to infection showed (organ-specific) enrichment in acute phase reactants. More specifically, these included haptoglobin in brain, and serum amyloid A proteins in kidney, liver and heart, which are both known to have predictive value for the diagnosis of systemic inflammatory processes (192). Methicillin-resistant *S. aureus* infection affected predominantly the liver vasculature, with a strong and tissue-specific increase in PRG4 levels (39). Although a role in sepsis has not been previously demonstrated, PRG4 has been established as an anti-inflammatory competitor for hyaluronic acid binding and it could therefore influence how immune cells interact with activated ECs (193). Besides, PRG4 has recently been suggested to serve as a potential therapeutic and biomarker in sepsis (194).

A follow-up study using vascular proteomics in the same model also showed a decrease in proteins involved in lipid metabolism in the liver, as well as a time-dependent procoagulant and antifibrinolytic shift of the endothelium. Interestingly, the changes in haemostatic proteins occurred well before signs of organ dysfunction and thrombosis were observed (38).

### Organotypic changes in sepsis: single cell RNA-seq

Organotypic changes in animal models of sepsis are now well-established, but the previous studies evaluated the EC population within an organ as a whole, which prohibits determining whether changes follow a distinct pattern along different branches of the vascular tree. Using scRNA-seq analysis, a recent study determined cell-specific changes in acute kidney injury during a 48 h course after LPS injection (195). Although this study did not specifically focus on the endothelium, they did notice that changes in ECs already started to appear 1 h post-LPS, whereas changes in expression profiles of proximal tubule epithelial cells took 4 h to occur. Moreover, based on gene regulatory networks and receptor-ligand crosstalk analyses, it was shown that there is general cell–cell communication failure around 16 h after LPS exposure, which plays not only a role in the progression to organ dysfunction, but also in activating recovery pathways (195).

A scRNA-seq study looking at CLP-induced changes in non-parenchymal cells of the liver was able to identify 4 distinct EC subpopulations, which were all uniquely affected by CLP (196). Out of these 4 subsets, 2 were nearly exclusively present under sepsis conditions. Although they both displayed a proinflammatory phenotype, they presented at different stages during infection, with expression data suggesting that one subset likely plays a role in EC activation while the other contributes to the adaptive immune response to infection as antigen presenting cells (196). Similarly, scRNA-seq of non-neuronal cells of the mouse cerebral cortex identified 6 main EC subsets under basal conditions, with an additional minor subpopulation of venous ECs characterized by the presence of *Icam-1* and *Vcam-1* (47). The significance of the latter was evaluated 2 h after LPS exposure, where based on immunohistochemical analyses it was suggested that this EC subpopulation predominantly exists in the postcapillary venules and may serve as an initial site for leukocyte recruitment. Finally, scRNA-seq of lung endothelium established 2 major EC populations under naïve conditions, with one subpopulation expressing immune response genes while the other expressed genes associated with development and regeneration (197). Evaluation of these subsets after LPS exposure showed distinct transcriptional changes in the early and late inflammatory response. Interestingly, during the recovery phase (3 days post-LPS) a third population of proliferative ECs emerged from the developmental subset, which is presumably important for vascular regeneration.

Taken together, these scRNA-seq studies have established several endothelial subsets per organ, which show unique properties depending on their location along the vascular tree as well as their function. Sepsis-induced transcriptional changes imply that these subpopulations participate in distinct but complementary tasks.

## EC heterogeneity in sepsis—molecular treasure trove and pharmacological Gordian knot

Because of the direct contact between the endothelium and blood, EC-derived proteins easily emerge in plasma, and can therefore be used as potential biomarkers. Over the years, hundreds of biomarkers in relation to sepsis for diagnostic and/or prognostic applications have been proposed, typically focusing on inflammation, coagulation, endothelial damage or vital organ function, although only a small fraction of these have been evaluated in larger patient cohorts or validated across multiple studies (198). Several of these are indeed EC-derived, and not surprisingly, they are often related to leukocyte adhesion, vascular permeability, or coagulation processes (Table 2). It is important to note, however, that despite the observed differences in plasma levels and their reported associations with disease severity, none of them (either alone or in combination) are truly specific or sensitive enough to accurately diagnose sepsis or predict sepsis outcome (198). Furthermore, many of these markers are expressed by ECs in multiple organs, and changes in blood levels may thus represent either general EC dysfunction or organ-specific EC damage. Especially the latter would be valuable for diagnostic and prognostic purposes since several studies have indicated that EC dysfunction precedes organ failure (195, 196). Therefore, being able to identify organotypic EC-derived proteins in the circulation could be used to predict organ dysfunction so preventive measures can be taken. Unbiased -omics studies using transcriptomics and proteomics analyses as described here could guide the identification of such biomarkers.

**Table 2:** Overview of EC-derived biomarkers and their main diagnostic/prognostic value based on selected references.^a^

Process	Biomarker	Main outcome	References
Cell adhesion	Soluble E-selectin	Increased levels associated with sepsis severity, organ dysfunction and mortality	(18, 118, 120)
	Soluble ICAM-1	Increased levels associated with sepsis severity, organ dysfunction and mortality	(18, 118, 120)
		Prognostic value for 90 days mortality in severe sepsis and septic shock	(123)
	Soluble VCAM-1	Increased levels associated with sepsis severity, organ dysfunction and mortality	(18, 118, 120)
		Prognostic value for 90 days mortality in severe sepsis and septic shock	(123)
		Increased levels in patients with SIRS, but not independently associated with it	(9)
Permeability	Angiopoeitin-2	Increased levels associated with mortality	(122)
	VEGFR1	Increased levels associated with sepsis severity, organ dysfunction and mortality	(18, 120, 122)
Coagulation	Soluble thrombomodulin	Early predictor multiple organ failure and mortality	(11, 12)
		Increased levels in non-survivors	(122, 123)
	PAI-1	Increased levels associated with sepsis severity, organ dysfunction and mortality	(18, 120, 176)
	VWF	Increased levels in non-survivors	(122)
		Increased levels in patients, but no correlation with disease severity, organ dysfunction or disease outcome	(8)

a A complete overview of biomarkers previously associated with sepsis diagnosis and/or prognosis is provided in reference (198).

Besides identifying biomarkers, -omics studies are also likely to generate information from which new candidate molecules for drug interventions can be derived, as the easy access and early responses of the endothelium provides a unique potential for therapeutic intervention. Historically, proposed treatments for sepsis have focused on inhibiting inflammation (e.g., anti-TNFα or IL-1Ra therapy), promoting vascular integrity (via TIE2 stabilization), or counteracting the procoagulant shift of the endothelium (e.g., by using activated protein C, or recombinant soluble thrombomodulin) (199–205). However, none of these have resulted in approved drugs currently used in the clinic. There are several plausible explanations for this (see also next section), including the now widely recognized organotypic EC heterogeneity which could have contributed to their lack of success, as many of these drugs might have been developed under the assumption that circulating biomarkers would represent a general dysfunctional EC phenotype. Based on current data, this is likely incorrect, and these early developed strategies may therefore cause more harm than good under certain conditions, as EC activation during inflammation is first and foremost a beneficial response aimed at maintaining tissue homeostasis by eliminating the pathogen and limiting damage (206).

It is important to obtain a comprehensive understanding of the differences in kinetics of EC activation and dysfunction across vascular beds, as well as the presence of unique molecules that can distinguish between affected EC subsets since this would support the development of targeted delivery approaches to limit unwanted side-effects. Trying to leverage EC heterogeneity for therapeutic gain, drug delivery approaches have employed antibodies coupled with therapeutic agents or targeted nanobodies against EC surface markers such as PECAM, E-selectin, and VCAM-1 (207–211). Yet, while intended to target dysfunctional ECs, in reality many non-dysfunctional ECs also express these markers and thus molecular information at a high cellular resolution will be required to establish specific cargo transfer of these drug delivery systems. Besides the decision on which receptor to target (Figure 3), the choice of drug treatment needs to be carefully considered to avoid that risks associated with treatment outweigh the benefits. Therefore, we need to get a better understanding of the kinetics of (downstream) intracellular pathways affected in sepsis, and their status in different microvascular beds. This pharmacological Gordian knot can in theory, and possibly also experimentally in the near future, be disentangled by combining transcriptomics and proteomics with, for example, protein kinase activity platform techniques (kinomics) to study activation status of multiple signal transduction cascades (212).

## Challenges and future directions

Despite all efforts to develop therapies to improve sepsis outcome, at present only supportive organ care can be offered to sepsis patients. This lack of pharmacologically effective treatment strategies to counteract the pathophysiological processes in sepsis is in stark contrast to the successes observed in preclinical studies that interfere with dedicated molecular pathways. The following part will discuss some of the challenges sepsis research has faced, and is still facing, that could explain this discrepancy, together with future directions to help move the sepsis field forward.

### Experimental models of sepsis

The complexity of sepsis requires the use of experimental animal models to study (cell-specific) mechanisms underlying pathophysiology. However, even in an intact animal such as the mouse, it is difficult to accurately recapitulate sepsis pathobiology. This is partly due to physiological differences between mice and humans, as well as their respective response to a septic insult, as mice are more resistant and show greater resilience, and are typically relatively young and healthy. Especially this latter is opposite from the “average” septic patient, who is generally older with more comorbidities (2). Furthermore, particularly in small animal models, it is not feasible to incorporate organ support that is typically given to sepsis patients.

A previous study compared 3 different murine sepsis models and showed clear differences in kinetics and magnitude of the inflammatory response (146). These data indicate that the model strongly influences the host response and findings from one model do not necessarily reflect general sepsis patho(physio)logy. Although sepsis models thus need to be carefully chosen based on the goal of the study, these differences can also be used to our advantage: in order to standardize preclinical studies, a systematic multi-model approach should be developed in which drug candidates are tested throughout models with greater levels of complexity (i.e., LPS followed by CLP in mice, followed by studies in bigger animal models in which care can be given as applied in the ICU) as research progresses. In addition, therapeutic interventions should also be tested at different time points after insult to fully address their pharmacological effectiveness when drugs are administered at different stages of sepsis progression, as each stage is likely associated with distinct changes in the host response. Furthermore, attention should be paid to pharmacokinetics/pharmacodynamics in these pre-clinical studies, to prohibit misinterpretation of treatment outcome due to differences in drug half-life and/or metabolism, for example. Addressing cell type-specific pharmacodynamics furthermore aids in understanding which effects of drug treatment occur in cells considered to be the target of therapeutic intervention versus cells that engage in the pathophysiology but are not target cells *per se*. scRNA-seq techniques as well as isolation of cellular subsets by laser microdissection prior to RNA-seq (213) or spatial transcriptomics, which allows evaluation of cellular interactions *in situ* and thus provides information on how the alterations in ECs affect underlying parenchyma, will be critical technological advancements for this purpose, and eventually create a full “pharmacolomics” view in which drug effects are related to pathophysiological changes. This approach is of crucial importance to make a rational decision to enter a clinical testing phase, and to provide substantial information to make a well-informed choice of biomarkers to measure in the restricted clinical samples available.

### Clinical challenges

A retrospective analysis of >20,000 sepsis patients identified 4 distinct sepsis phenotypes that correlated with host response patterns and clinical outcome (214). Interestingly, this study suggested that including patients in trials based on the specific sepsis phenotype could lead to drastic differences in outcome depending on the clinical intervention. For example, they showed that treatment with activated protein C (Xigris) which, despite its dual action as an anticoagulant and involvement in protecting the vascular barrier, was discontinued as a therapy for sepsis due to an increased bleeding risk (203, 215), would improve outcome in patients with sepsis-induced disseminated intravascular coagulopathy whereas for other phenotypes it was indicated to be harmful (214). A similar result was described in a reanalysis of sepsis patients treated with recombinant thrombomodulin, which was shown to only benefit patients with a severe coagulopathy phenotype (216). These data illustrate that unbiased patient inclusion can cause conflicting results as interventions can be beneficial or detrimental to certain patient populations. Therefore, it has been suggested to revisit previous sepsis treatments that failed in clinical trials, and re-evaluate them in better defined patient groups.

Another clinical challenge pertains to the use of biomarkers to guide clinical decisions. Although a consistent difference in biomarker patterns was observed between sepsis phenotypes (214), it required data obtained from several biomarkers as not one by itself is specific or sensitive enough to diagnose sepsis reliably. Besides their lack of specificity regarding cellular and organ origin, biomarker levels display large inter-patient variability and they are time-dependent as they fluctuate based on disease progression. Thus, the absence of a significantly elevated protein in serum does not automatically preclude sepsis diagnosis, and it is therefore important to continuously monitor patients to accurately assess the pathophysiological stage of sepsis, starting as soon as the patient presents with clinical symptoms through at least until they get discharged from the ICU. This is not only to initially determine the course of sepsis, but also to monitor effects of organ support, and possibly drug treatment regimens.

### Integration of -omics studies

Based on their early response, ECs have been consistently suggested as a target for therapeutics. Even though better biomarkers and patient stratification will be helpful in determining a treatment plan, it is unlikely that targeting only one molecule or pathway will be sufficient to ensure vascular protection and facilitate restoration of EC function since the molecular mechanisms underlying sepsis are so complex. This complexity has become even more fully appreciated with the introduction of unbiased -omics approaches.

In light of the latter, it is interesting to take a closer look at the efforts taken to study SARS-CoV-2 infection leading to COVID-19. Once the true impact of this disease became apparent, the scientific community quickly rallied to employ methods such as (single cell) RNA-seq and spatial transcriptomics to generate testable hypotheses leading to a better understanding of the molecular mechanisms driving the host response and general patho(physio)logy of this devastating disease (90, 217–222). Although several parallels can be drawn between COVID-19 and sepsis, including the presence of endotheliopathy (219, 221), it is interesting to note that studies using these relatively new technologies have only started to emerge recently in the sepsis field (195, 196). Nonetheless, these are expected to make a great stride forward in elucidating the complexity of sepsis.

Traditional transcriptomics approaches are invaluable for understanding the dynamics of gene expression, but they do not tell the full story. miRNAs, including EC-derived miRNAs, are increasingly recognized for their role in sepsis and potential as biomarker (223–226). Although studying miRNAs in an unbiased manner has been historically more difficult due to technical limitations (227), our recent studies combining RNA-seq and miRNA-seq data obtained from distinct microvascular segments in the kidney have demonstrated its potential to predict RNA-miRNA relations in the complexity of the vasculature in an *in vivo* setting (213). Thus, these data could serve as a starting point for the identification of targets for therapeutic interventions. Furthermore, epigenetic regulation of gene expression and the resulting proteome also provide important information, particularly the proteome of the vessel wall (38, 39) and the plasma proteome as these could lead to the identification of markers used for therapeutics or diagnostic/prognostic purposes. While a few translational research teams have actively sought to create a biobank of biological materials including plasma, serum, urine, and tissue samples obtained from sepsis patients, a broad concerted strategy that can be incorporated into the daily workflow of clinical departments is at present lacking. In addition, miniaturization of -omics analyses is key to make the desired progress in unravelling the molecular basis of sepsis and sepsis-related multiple organ dysfunction and to make -omics not compete with standard assessment of clinically relevant parameters but become an add-on assessment instead (112). Even though it is currently not feasible (yet) to base a treatment plan on -omics data generated from patient samples given its relatively slow turn-around time whereas sepsis rapidly progresses, eventually the integration of -omics data will be required to map the spatiotemporal changes occurring during sepsis progression. This will create opportunities for a systems biology approach to get a holistic picture of sepsis pathophysiology required for developing rational therapeutic intervention strategies.

## Conclusion

Endothelial cells are often referred to as gatekeepers of tissue homeostasis given their unique position between the blood and parenchyma, and their interspersed distribution throughout the body. This makes ECs a particularly relevant cell type for therapeutic strategies since they are not only early responders and active contributors to disease pathophysiology, including sepsis, but are also easily accessible. However, their heterogeneity across vascular beds under both physiological and pathological conditions makes this notion more complicated and may explain, at least partly, the lack of success in our quest of developing effective treatment options for sepsis aimed at the endothelium. The introduction of -omics approaches has enabled the field to get a better understanding of organ- and (endothelial) cell-specific changes that occur during sepsis progression. This has not only contributed to our general knowledge of pathogen-host interactions, but it is also expected that it will open the doors for identifying new therapeutic targets, molecules for targeted drug delivery, and biomarkers with diagnostic, prognostic and/or predictive value. Together with patient stratification, -omics data from pathophysiological and pharmacological studies is expected to unveil the molecular treasure trove to advance disentangling, or maybe even cutting, the Gordian knot of EC heterogeneity in the contribution to sepsis pathology.

## Author contributions

AC and GM developed the concept of the article and performed literature searches. Both authors contributed to the article and approved the submitted version.
